# COVID-19 vaccination and psychological status of Iranian dental students

**DOI:** 10.3389/fpubh.2022.946408

**Published:** 2022-09-14

**Authors:** Hannaneh Ghadirian, Mohammad Reza Khami, Seyyedeh Niloufar Tabatabaei, Amir Hossein mirhashemi, Rashin Bahrami

**Affiliations:** ^1^Department of Orthodontics, School of Dentistry, Tehran University of Medical Sciences, Tehran, Iran; ^2^Research Center for Caries Prevention, Dentistry Research Institute, Community Oral Health Department, School of Dentistry, Tehran University of Medical Sciences, Tehran, Iran

**Keywords:** COVID-19, temporomandibular joint disorders, depression, sleep, vaccines

## Abstract

**Objective:**

The purpose of the present study was to investigate the association of COVID-19 vaccination with the quality and quantity of sleep, the level of stress, and temporomandibular joint (TMJ) disorders (TMDs) in Iranian dental students.

**Materials and methods:**

In this cross-sectional research, we applied a questionnaire including 30 questions on the Perceived Stress Scale (PSS), sleep quality and quantity, Diagnostic Criteria for Temporomandibular Disorders (DC/TMD), and vaccination status. All vaccinated students of the dental schools located in the city of Tehran were invited to participate in the study. Participants were divided into three groups: those vaccinated for less than a month, those vaccinated for 1–3 months, and those vaccinated for more than 3 months. A paired *t*-test served for statistical analysis.

**Results:**

Overall, 171 out of 235 students (72.77%) completed the questionnaire, among which 90 individuals were fully vaccinated, and were included in the data analysis. Stress levels decreased (mean difference = −1.23, *p*-value = 0.002) and sleep quality and quantity improved mostly 1–3 months after the vaccination (mean difference = −0.5, *p*-value = 0.016). However, TMD symptoms were mostly alleviated in people vaccinated for more than 3 months (mean difference = −2.86, *p*-value <0.05). In this respect, no significant difference was observed between the two genders.

**Conclusion:**

According to the results of the study, vaccination was associated with the improvement of psychological consequences of the COVID-19 pandemic. It is recommended that further longitudinal studies be conducted on larger sample sizes and different age groups by using various data collection methods (especially regarding the assessment of TMD).

## Introduction

The COVID-19 epidemic has changed the daily plans of people and has caused concerns regarding their health and welfare (1). Similar to other countries, preventive measures, such as social distancing, travel bans, school closures, and changing lifestyles and business methods have been implemented in Iran to decrease disease transmission. Due to the current pandemic, gatherings in public places, such as gyms and parks that allowed people to exercise have been banned from applying social distancing. Several studies have shown that reduced physical activity negatively affects the psychological condition of individuals (2, 3). In addition, it causes depression, stress, and anxiety, along with reduced sleep quality (4). Quarantine, changes in habits, sleep disorders, and increased levels of depression, anxiety, and stress associated with the COVID-19 epidemic have significantly negative impacts on people's lives (5). In this respect, two adverse effects are temporomandibular joint (TMJ) disorder (TMD) and bruxism, which are highly correlated with stress. In a study, Carrillo-Diaz et al. evaluated the prevalence of parafunctional habits and bruxism in adolescents in two periods before (T1) and after (T2) COVID-19 pandemic. Their results indicated increased bruxism during T2, compared with T1, due to stress caused by the COVID-19 pandemic (6).

Meanwhile, TMD occurrence depends on multiple factors. Studies show that stress and anxiety levels and sleep disorders are among the TMD risk factors (7, 8). In addition, since low sleep quality affects patients' quality of life and health status, it could cause TMD (9, 10). Numerous studies have reported the relationship between TMD and sleep disorders (11, 12).

Healthcare centers, such as dentistry schools, have served people during the pandemic. Long hours of activity in extremely stressful environments are associated with various psychological outcomes. Academic tasks, personal problems, the environment, and economic situations increase stress in dental students. In today's conditions, worries about infection, increased numbers of people with the disease, fear of death, lack of information, and misinformation about the COVID-19 disease have created a more stressful atmosphere that may even affect the quality of sleep and daily activities (13, 14). Moreover, people who are quarantined often feel lonely and angry because of losing their social communication (15). High stress and anxiety levels have been observed in healthcare personnel, mostly women (16, 17).

Fortunately, the COVID-19 vaccine has become widely available recently, and medical staff, such as university students, have been vaccinated. Given the vaccine's prophylactic effect, the question is whether it also improves the psychological conditions imposed by the pandemic. In different populations, some studies indicate a big difference in the outbreak of TMD and its symptoms (18–20). The reason could be the difference in designs of studies, measurement tools, and methods for diagnosing TMDs (21).

The Diagnostic Criteria for Temporomandibular Disorders (DC/TMDs) can be used to diagnose TMD as a reliable and convenient measure (22, 23). The Pittsburgh Sleep Quality Index (PSQI) has been widely used to evaluate the quality of sleep as a reliable tool (24–26). The Perceived Stress Scale (PSS) can be used to evaluate the level of stress (27). All these tools have been indicated to have good validity and reliability in the Iranian population (27–29).

The purpose of the present study was to investigate the association of COVID-19 vaccination with the quality and quantity of sleep, the level of stress, and TMDs in Iranian dental students.

## Method and material

### Study design

This was a cross-sectional study performed on vaccinated dental students of the dental schools located in Tehran, Iran, who were in the clinical phase of their dentistry program (year 4, 5, and 6 of the 6-year Doctor of Dental Surgery or DDS program). This study was done from May to July 2021.

### Study size

According to α = 0.05, β = 0.2, standard deviation (SD) = 9.52, and power = 80%, the minimum sample size was calculated to be 30 samples in each group (<1 month, 1–3 months, and more than 3 months).

### Ethical statement

The study protocol was reviewed and approved by the institutional Ethics Committee of the School of Dentistry, Tehran University of Medical Sciences (Ethical Approval number IR.TUMS.DENTISTRY.REC.1400.163).

The research objectives were explained to all participants both orally and in written form, and informed consent was obtained from all participants. The respondents were assured of the confidentiality terms regarding their personal information. Notably, participation was voluntary, meaning that subjects were allowed to withdraw from the research at any time.

### Inclusion and exclusion criteria

The inclusion criteria included dental students who were fully vaccinated (two doses) and passed at least 2 weeks after the final dose because the vaccine requires a minimum of 14 days to create immunity against the virus (30), who were single, over the age of 18 years, who were Persian speaker, and who had no physical and mental diseases. All participants had to be vaccinated with the same vaccine.

The exclusion criteria included students who were not fully vaccinated or not meeting the inclusion criteria, any students with a history of TMJ trauma and/or any TMJ surgical interventions, and students who were under treatment for anxiety and/or depression.

### Data collection method

In the present study, data were collected using a questionnaire comprising demographic characteristics (age, gender, marital status, medical history, and time passed since receiving the vaccine), a Perceived Stress Scale (PSS), Diagnostic Criteria for Temporomandibular Disorders (DC/TMDs), and selected questions from the Pittsburgh Sleep Quality Index (PSQI) regarding sleep quality and quantity.

#### Stress

The PSS is one of the most applicable scales to assess mental pressure caused by stress (psychological stress) (31). The tool, which encompasses 10 items, was used in the current study to compare the thoughts and feelings of the subjects at two different times (before and after vaccination). In addition, respondents were asked about changes in the frequency of the occurrence of emotion in each question.

#### Temporomandibular disorder

The Persian version of the DC/TMD includes questions about TMD symptoms (28). Its items have been designed in line with a screening of TMDs. The tool involves 10 items about joint and facial muscle pain, headaches, jaw sounds, jaws that lock in the open- or closed-mouth position, and average pain intensity. The questions in this section were in the form of changes in the symptoms shown in the two time periods before and after vaccination (26, 32).

#### Sleep quality and quantity disorders

Six items of the PSQI were selected to evaluate the sleep quality and quantity of the subjects by comparing these variables in two different periods (before and after vaccination).

In the end, four questions related to the overall effect of vaccination on stress level, temporomandibular joint, and sleep quality and quantity were added to complete the questionnaire. The items were scored based on five alternatives: increased (score 0), no change (score 1), decreased (score 2), never (before or after vaccination, has not had this feeling) (score 3), and no comment (score 4).

### Participants' matching process

The matching method was used to remove the confounding effect; this is done by ensuring an equal distribution of the confounders (age, sex, city, dorm…) among groups (<1 month, 1–3 months, and more than 3 months).

### Statically analysis

Descriptive and inferential statistics were performed using the Statistical Package for the Social Sciences version 23.0 software (SPSS Inc., Chicago, Illinois, USA). Two-sample *t*-tests were used to detect significant differences between the groups at each assessment time. Repeated-measures analysis of variance (ANOVA) was used to detect significant differences between the three assessment times in each group. The significance level was set at 0.05.

## Results

**Figure 1:**
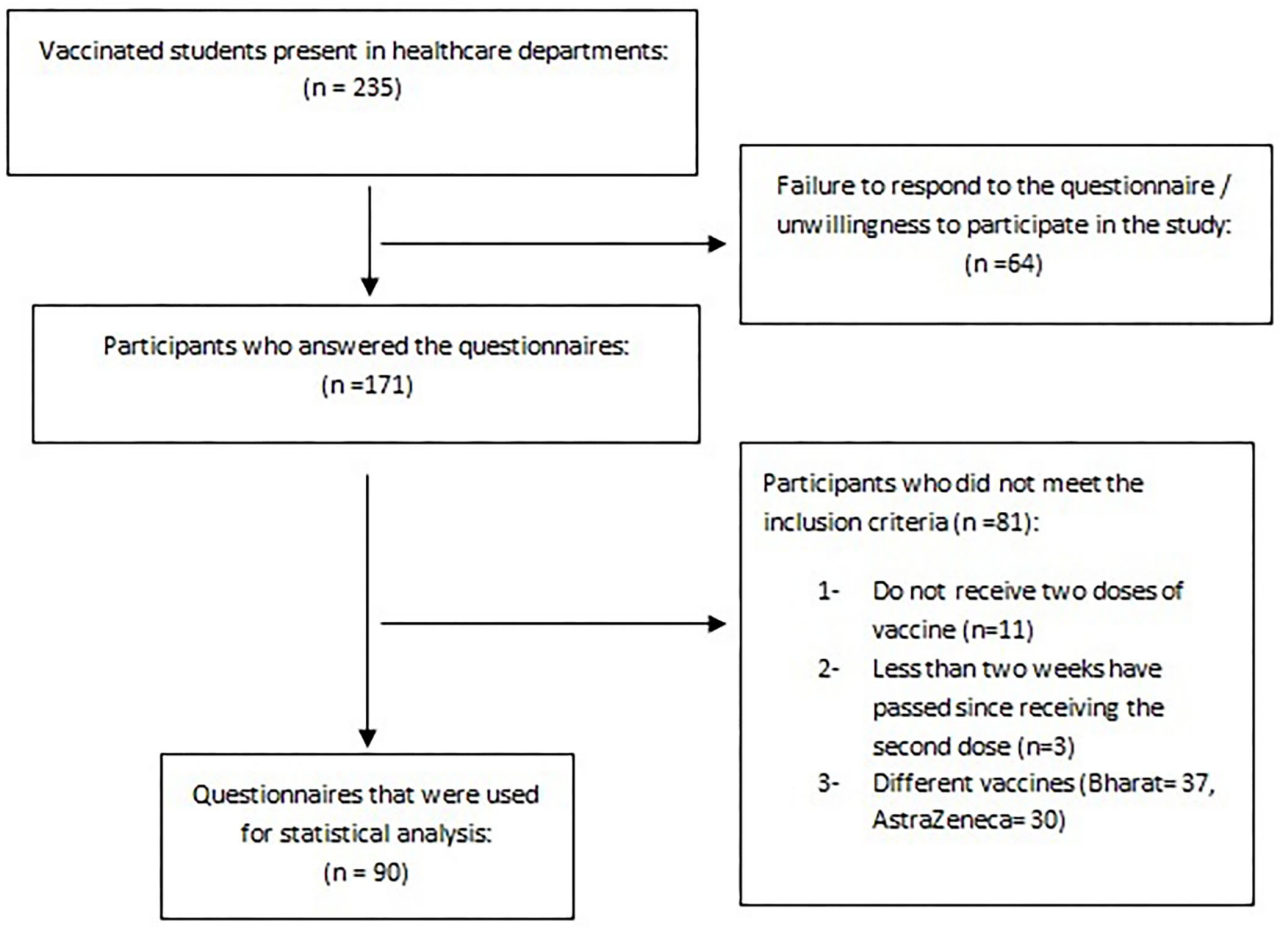
Selection of the final sample.

The questionnaire was distributed among 235 vaccinated dental students. Of these students, 72.77% (171 students) filled out the questionnaires; of which 90 participants fulfilled inclusion criteria and had received one similar type of vaccine (Sinopharm, Beijing, China). Figure 1 shows how the final sample was selected. In general, 48 men and 32 women participated in the three groups based on a uniform distribution. The age average of participants was 24.82 ± 1.28 years old.

### Perceived stress

Table 1 represents the overall score of perceived stress within three intervals of <1 month after the second vaccine dose injection, 1–3 months after injection, and more than 3 months after injection, according to gender; there was a significant reduction in the overall score of stress in all three studied time intervals (mean difference = −1.23, −4.10, and −2.73, respectively). The highest reduction rate of stress was seen in the time interval of 1–3 months after injection of the second vaccine dose; however, the reduction difference between the two genders was insignificant (*p*–value = 0.68).

**Table 1:** Overall stress score, temporomandibular disorder (TMD), and quality and quantity of sleep by time and gender (*t*-test).

**Time passed from the second vaccine dose / Gender**	**Less than 1 month**			**1–3 months**			**More than 3 months**			**Gender**	
**Variable**	**T**	**Sig**	**Mean**	**T**	**Sig**	**Mean**	**T**	**Sig**	**Mean**	**T**	**Sig**
			**difference**			**difference**			**difference**		
Stress.total	−3.45	0.002	−1.23	−7.69	0.000	−4.10	−7.18	0.000	−2.73	−0.417	0.67
TMD.total	−3.57	0.002	−1.43	−4.76	0.000	−2.10	−6.08	0.000	−2.85	−0.231	0.82
Sleep	−2.55	0.016	−0.50	−6.81	0.000	−1.80	−4.66	0.000	−1.00	−0.347	0.73

Table 2 shows the answers given to each PSS item. Improvements in self-confidence, emotion control, the management of important issues in the workplace, and anger control were observed in individuals who had received the second dose of the vaccine 1 month before starting the study. There was a significant decline in perceived stress levels within time intervals of 1–3 and more than 3 months after vaccination. Although there was an improvement in the management of important issues in the workplace 3 months after vaccination, the difference was insignificant.

**Table 2:** COVID−19 and questionnaire (*t*–test).

**Variable**	**Questions and answers**	**Less than 1 month**		**1–3 months**		**More than 3 months**	
		**Mean**	***p*–**	**Mean**	***p*–**	**Mean**	***P*–**
		**difference**	**value**	**difference**	**value**	**difference**	**value**
Perceived stress scale(modified version)	1–Number of the times you have felt anxious and inconvenient about unplanned events nowadays compared to the days before vaccination…	−0.81	0.42	−2.52	0.02*	−3.81	0.001*
	2–Number of the times you have felt not having control over important issues in your workplace nowadays compared to the days before vaccination…	−2.41	0.02*	−5.11	0.000*	−1.16	0.25
	3–Number of the times you have felt agitated and worried nowadays compared to the days before vaccination…	−1.14	0.26	−4.71	0.000*	−3.01	0.005*
	4–Number of the times you have felt that you don't have enough self–confidence nowadays compared to the days before vaccination…	−2.41	0.023*	−3.34	0.002*	−2.26	0.031*
	5–Number of the times you have felt that things don't take place according to your desire nowadays compared to the days before vaccination…	−1.14	0.26	−4.71	0.000*	−3.25	0.003*
	6–Number of the times you haven't been able to handle your routine works nowadays compared to the days before vaccination…	−0.81	0.42	−2.52	0.02*	−3.81	0.001*
	7–Number of the times you couldn't control your emotions nowadays compared to the days before vaccination…	−2.69	0.01*	−6.16	0.000*	−3.25	0.003*
	8–Number of the times you have felt that the people around pay attention to you nowadays compared to the days before vaccination…	−1.14	0.26	−4.71	0.000*	−3.52	0.001*
	9–Number of the times you have gone crazy because of some events that were out of your control nowadays compared to the days before vaccination…	−2.41	0.02*	−3.34	0.002*	−2.26	0.03*
	10–Number of the times you have felt that you aren't able to overcome the problems nowadays compared to the days before vaccination…	−1.14	0.26	−4.71	0.000*	−3.25	0.003*
Sleep quality and quantity evaluation	11–Number of the times you have felt tired at the time of waking up from sleep nowadays compared to the days before vaccination…	−0.81	0.42	−3.26	0.003*	−3.81	0.001*
	12–Number of the times you have had difficulty staying awake while driving, eating food, or nowadays compared to the days before vaccination participating in social activities…	−2.69	0.01*	−5.11	0.000*	−1.16	0.25
	13–Number of the times you have had a problem going to sleep nowadays compared to the days before vaccination…	−0.81	0.42	−4.71	0.000*	−3.01	0.005*
	14–Number of the times you have had a problem in staying energized while attending the faculty and the hospital departments nowadays compared to the days before vaccination…	−2.41	0.023*	−3.89	0.001*	−2.26	0.03*
	15–Number of the times you have needed to use medicine (prescribed or unprescribed) for going to sleep nowadays compared to the days before vaccination…	−1.14	0.26	−4.71	0.000*	−3.25	0.003*
	16–Amount (hours) of your sleep nowadays compared to the days before vaccination…	1.79	0.08	3.61	0.001*	2.80	0.009*
Screening of TMD and comorbidities (short and modified version)	17–Number of the times you have felt pain in your jaw or facial muscles nowadays compared to the days before vaccination…	0	1	−1.31	0.20	−1.79	0.08
	18–Number of the times you have heard a sound from your jaw joint while moving or using it nowadays compared to the days before vaccination…	−1.27	0.21	−0.63	0.53	−1.54	0.13
	19–Number of the times you haven't been able to open your mouth due to your jaw being locked nowadays compared to the days before vaccination…	0.81	0.42	−1.72	0.09	−1.72	0.09
	20–Number of the times you haven't been able to close your mouth due to your jaw being locked after opening your mouth nowadays compared to the days before vaccination…	−0.90	0.37	−1.99	0.06	−3.89	0.001*
	21–Number of the times you have felt pain in your neck nowadays compared to the days before vaccination…	0.70	0.49	−0.37	0.71	−0.90	0.37
	22–Number of the times you have heard buzzing in your ear nowadays compared to the days before vaccination…	1.14	0.26	−0.37	0.71	−1.98	0.06
	23–Number of the times you have had vertigo or dizziness nowadays compared to the days before vaccination…	1.68	0.10	0.44	0.66	−2.69	0.01*
	24–Number of the times you have squeezed or ground your teeth together nowadays compared to the days before vaccination…	−0.77	0.49	1.14	0.26	−1.54	0.13

^*^The mean difference is significant at the 0.05 level.

### Sleep quantity and quality

According to Table 1, there was a significant reduction in sleep disorders in all three intervals; however, the most improved sleep quantity and quality were observed 1–3 months after the injection of the second vaccine dose (mean difference = −1.80). The two genders differed insignificantly in this regards (*p*-value = 0.73).

According to Table 2, there was a significant reduction in hypersomnia and difficulty in staying energized throughout the day 1 month after full vaccination (*p*-value = 0.01). In addition, this table indicates significant mitigation in sleep disorders (insomnia) within all intervals.

### Temporomandibular disorder

According to Table 1, there was an improvement in TMD symptoms within time intervals, although the highest reduction was observed in individuals who had received the vaccine more than 3 months ago (mean difference = −2.85). There was no significant difference in TMD symptoms between the two genders (*p*-value = 0.82).

There was a significant reduction in jaw locking (*p*-value = 0.001) and feeling confused (*p*-value = 0.01) more than 3 months after vaccination (Table 2). However, no significant change existed 3 months after vaccination.

According to Table 3, among the individuals who had pain in their jaws or facial muscles within all of the time intervals after vaccination, the highest probability of pain was related to some regular jaw functions, such as pressing teeth together, chewing food, or chewing gum (67.8%).

**Table 3:** The prevalence of pain in the jaw or facial muscles based on the relevant function.

**Question**	**Answer**	**Less than 1 month**	**1 to 3 months**	**More than 3 months**	**Total**
Chewing hard or tough food	Yes	5	7	7	19
		25.0%	33.3%	38.9%	32.2%
	No	15	14	11	40
		75.0%	66.7%	61.1%	67.8%
Opening your mouth or moving your jaw forward to the other side	Yes	9	6	7	22
		42.9%	28.6%	38.9%	36.7%
	No	12	15	11	38
		57.1%	71.4%	61.1%	63.3%
Jaw habits such as clenching, grinding or chewing gum	Yes	14	13	13	40
		70.0%	61.9%	72.2%	67.8%
	No	6	8	5	19
		30.0%	38.1%	27.8%	32.2%
Other jaw activities such as talking, kissing, yawning	Yes	9	10	7	26
		45.0%	47.6%	38.9%	44.1%
	No	11	11	11	33
		55.0%	52.4%	61.1%	55.9%

### The overall effect of vaccination on stress level, TMJ, sleep quantity, and quality

Table 4 presents the responses to the questions regarding the overall effect of vaccination on stress level, TMJ, and sleep quality and quality. The highest reported effect of vaccination on stress level was observed in 1–3 months after vaccination, and the majority of participants in the time interval of 1–3 months obtained the highest score which was 4 (12 out of 30, 40%).

**Table 4:** COVID−19 vaccination impact on stress level as self–evaluation.

**Time passed from the second vaccine dose**	**Variable**	**Score**				
		**0**	**1**	**2**	**3**	**4**
Less than 1 month	Stress. Total	6.7%	23.3%	40.0%	13.3%	16.7%
	TMD. Total	23.3%	50.0%	23.3%	3.3%	0.0%
	Sleep quality. Total	13.3%	40.0%	30.0%	16.7%	0.0%
	Sleep quantity. Total	16.7%	46.7%	30.0%	6.7%	0.0%
1 to 3 months	Stress. Total	0.0%	0.0%	20.0%	40.0%	40.0%
	TMD. Total	26.7%	36.7%	23.3%	10.0%	3.3%
	Sleep quality. Total	0.0%	23.3%	63.3%	10.0%	3.3%
	Sleep quantity. Total	6.7%	56.7%	33.3%	3.3%	0.0%
More than 3 months	Stress. Total	23.3%	36.7%	30.0%	6.7%	3.3%
	TMD. Total	10.0%	16.7%	23.3%	36.7%	13.3%
	Sleep quality. Total	13.3%	43.3%	43.3%	0.0%	0.0%
	Sleep quantity. Total	6.7%	56.7%	33.3%	3.3%	0.0%

The highest reported effect of vaccination on TMJ was observed more than 3 months after vaccination. The highest score in this group equaled at 3 (11 out of 30, 36.7%).

The highest reported effect of vaccination on sleep quantity and quality was observed 1–3 months after vaccination. According to results, the highest percentages equaled score 2 for sleep quality (19 participants), and scores 3 and 4 for sleep quantity (12 participants).

## Discussion

The respiratory system is the common transmission way of COVID-19 infection. Because of the enormous amount of aerosol, dental clinics and universities are the riskiest places (33). The uncertainty and unpredictability of this virus cause the world to face a vast and stressful problem. Additionally, students during the pandemic and peak of coronavirus disease 2019 were active in universities, which caused a lot of stress for them. For this reason, previous studies have examined the psychological effects of the COVID-19 pandemic on dental students; their studies have shown that stress levels and their side effects, such as decreased sleep quality and quantity, have increased in these students following the COVID-19 pandemic (34–36).

The main objective of the present study was to evaluate the association of vaccination against COVID-19 with sleep quality and quantity, and stress level, as well as its relationship with temporomandibular joint disorders among Iranian dental students. According to our hypothesis, vaccination has a positive effect on improving the quality and quantity of sleep, level of stress, and temporomandibular joint disorders of dental students. This placebo effect has been reported for other medications as well. For example, studies have shown that placebo drugs had a similar positive impact to antidepressants; however, they did not have the side effects of antidepressant medications. Thus, they advised using it before prescribing antidepressant drugs (37). Regarding vaccination against COVID-19, there was just one study available that evaluated the effect of COVID-19 vaccination on the anxiety levels of dental professionals; their results showed the positive impacts of vaccination on the anxiety levels (38).

The response rate was 77.72% in the present study (171 from 235 participants), among which 90 participants met the study inclusion criteria and received the same vaccine (Sinopharm, Beijing, China). The results of the present study show that vaccination was associated with a reduction in perceived stress levels, with improved TMD symptoms, and with quality and quantity of sleep. We found the maximum improvement in the quality and quantity of sleep with a reduction in stress levels after 1–3 months of receiving the second dose of the vaccine. The reduction in stress levels after 3 months was more than in the other two periods. However, this reduction was lower compared to that in the period of 1–3 months after receiving the vaccine. In other words, the psychological effects were more pronounced 1–3 months after the injection. No significant difference was observed between the two genders in this regard.

Temporomandibular disorder includes a group of clinical alterations to the masseter muscles, temporomandibular joint, and its structures. It is estimated that 50–70% of the world population show TMD signs and symptoms at some stages of life (39). The prevalence of TMD is common among dental students due to job-related stresses (40). Stressful conditions lead to increased activity of masseter muscles that causes temporomandibular joint structure change, which clinically results in pain, functional limitations, and finally negative impact on the quality of life (41, 42).

Similarly, Gaş et al. study reported increased TMD, reduced sleep quality, and increased levels of depression, anxiety, and stress among dentistry students in Turkey during the COVID-19 pandemic (43). Medeiros et al. (44) conducted a study to evaluate the prevalence of TMD, anxiety, and depression among dentistry students during social isolation due to the COVID-19 disease. Their study showed that COVID-19 social isolation was in relation to an increased prevalence of TMD symptoms, anxiety, and depression (44).

Adequate quality sleep is vital for the immune system. Studies regarding sleep and COVID-19 vaccination are not yet available, though studies on other vaccines showed reduced efficacy due to sleep deprivation (45). Sleep affects different safety factors, and sufficient sleep is associated with a decreased risk of infection, healing of infection, and better vaccination efficacy. On the other hand, sleep deprivation disrupts how the body protects itself since white blood cells (immune system cells), which travel to the damaged part of the body, can be reduced (46). Sleep deprivation reduces the immune response through T cells, which are active participants in immune responses. Lack of sufficient sleep affects the immune response that increases stress hormone levels, which decreases the integrin level, a molecule that helps T cells bond to virus-infected cells (47, 48).

Stress can disrupt the immune system's function and even change the response to vaccination. In a study on BSc students, social dysfunction predicted low antibody levels following the conjugated meningitis C vaccine (49). However, simple solutions can increase the effectiveness of the COVID-19 vaccine in people with stress. In this regard, a solution is to perform high-intensity exercises and sufficient sleep for 24 h before vaccination. This allows the immune system to operate at peak performance and guarantees the best and strongest immune reaction in the fastest time possible (50).

In a study, Hall et al. showed higher stress levels and sleep disorders in female participants (30). In the current research, however, no significant difference was observed between the two genders regarding improvement following vaccination. In other words, assurance of the decreased possibility of disease transmission following vaccination was not related to gender.

Following vaccination, a reduction in TMD symptoms was observed in three study periods. A jaw or facial muscle pain, jaw locking, neck pain, tinnitus, dizziness, and bruxism showed no significant change in the 1st month after injection; there was even a slight increase in the jaw or facial muscle pain, jaw locking after mouth closing, neck pain, tinnitus, and dizziness in the 1st month after injection. Within 1–3 months after vaccination, we saw a non-significant reduction in these symptoms (except for bruxism and dizziness). Students who had been vaccinated for more than 3 months had all the signs of a declining trend; in particular, the jaw locking and the feeling of dizziness significantly reduced. The later onset of a declining trend in TMD symptoms may be related to its association with stress. Although the efficacy of existing vaccines remains questionable, the relative safety of the resulting immunity appears to have helped reduce stress levels, sleep disorders, and TMD symptoms.

Studies available in connection with the coronavirus vaccine evaluate the effect of stress on its effectiveness or the stress of possible side effects of the vaccine (51, 52); but so far no study has looked at how the stress, TMD, and sleep disorders caused by the pandemic has changed since receiving the vaccine and reducing the risk of transmitting the disease. However, the present study was not without limitations, but efforts were made to minimize bias; for example, to neutralize the effect of confounders, the participants' matching process was used.

One strong point of the present study is its good response rate (77.72%). The current study is the first research that finds an association among sleep quality and quantity, stress level, and TMD (DC/TMD criteria) in vaccinated (COVID-19) and unvaccinated dental students. However, certain limitations do exist. The self-report questionnaire is among the limitations of the present study. Previous studies have shown its limitations in evaluating temporomandibular joint disorders, as some participants may feel that they should give the appropriate or rational response. Moreover, clinical evaluations help detect TMD symptoms. The present study is a cross-sectional study, though longitudinal studies are superior to this study, in which the questionnaires are handed to the participants at different points of time for better analysis of the findings. Comparing different vaccines was not feasible in this study due to inadequate access.

## Conclusion

The current study suggests that vaccination is associated with improving the quality and quantity of sleep, stress level, and temporomandibular joint disorders of dental students. It is suggested that further studies are required to be conducted with higher sample sizes on different age groups using other data gathering methods, particularly regarding TMD evaluation.

## Data availability statement

The original contributions presented in the study are included in the article/supplementary material, further inquiries can be directed to the corresponding author.

## Ethics statement

The studies involving human participants were reviewed and approved by Ethics Committee of School of Dentistry, Tehran University of Medical Sciences (Ethical Approval number IR.TUMS.DENTISTRY.REC.1400.163). Written informed consent for participation was not required for this study in accordance with the national legislation and the institutional requirements.

## Author contributions

HG and RB: methodology. HG: editing and project administration. RB: writing—review and editing, writing—original draft, and conceptualization. MK: revision and review and editing. SN and AM: investigation, questionnaire distribution, and writing. All authors contributed to the article and approved the submitted version.

## Conflict of interest

The authors declare that the research was conducted in the absence of any commercial or financial relationships that could be construed as a potential conflict of interest.

## Publisher's note

All claims expressed in this article are solely those of the authors and do not necessarily represent those of their affiliated organizations, or those of the publisher, the editors and the reviewers. Any product that may be evaluated in this article, or claim that may be made by its manufacturer, is not guaranteed or endorsed by the publisher.
